# Assessment of the Relationship Between Haller Cells, Accessory Maxillary Ostium, and Maxillary Sinus Pathologies: A Cross-Sectional CBCT Study

**DOI:** 10.3390/diagnostics15202557

**Published:** 2025-10-10

**Authors:** İsmail Çapar, Çiğdem Şeker, Orhan Cicek

**Affiliations:** 1 Department of Dentomaxillofacial Radiology, Faculty of Dentistry, Zonguldak Bülent Ecevit University, Zonguldak 67600, Türkiye; isosmile@hotmail.com; 2 Department of Orthodontics, Faculty of Dentistry, Zonguldak Bülent Ecevit University, Zonguldak 67600, Türkiye; orhancicek@beun.edu.tr

**Keywords:** Haller cell, accessory maxillary ostium, ostium narrowing, ostium obstruction, maxillary sinus pathology, otorhinolaryngological, cone-beam computed tomography

## Abstract

**Background/Objectives**: Sinonasal anatomical variations, particularly Haller cells (HCs) and the accessory maxillary ostium (AMO), are critical structural factors that may increase surgical risks in dental and otorhinolaryngological (ENT) procedures and predispose individuals to chronic sinusitis. This study aimed to investigate the relationship between HCs, AMO dimensions, maxillary sinus ostium, and sinus pathologies using cone-beam computed tomography (CBCT). **Methods:** In this cross-sectional retrospective study, CBCT images of 443 patients (226 males, mean age 48.4 ± 15.4 years; 217 females, mean age 46.1 ± 15.2 years) were analyzed. The presence of HCs, AMO, ostium narrowing, and ostium obstruction were recorded, along with ostium dimensions. Relationships between these variations and sinus pathologies were statistically evaluated, with a *p*-value < 0.05 considered significant. **Results:** HC prevalence was 34.5% on the right and 39.5% on the left, while AMO was present in 39.5% on the right and 34.5% on the left. Bilateral AMO was significantly associated with localized mucosal thickening, and partial opacification was more common in cases with ostium obstruction. Significant relationships were observed between HC presence and ostium narrowing. While HCs and ostium narrowing did not significantly influence maxillary sinus pathologies, sex (right OR = 0.335; left OR = 0.384; *p* < 0.001) and the AMO (right OR = 1.698, *p* = 0.018; left OR = 1.713, *p* = 0.014) were found to have a significant impact. **Conclusions:** It was concluded that (i) HCs may contribute to ostium narrowing and impaired sinus drainage, thereby increasing the risk of chronic sinusitis; (ii) the presence of a bilateral AMO is strongly associated with localized mucosal thickening; (iii) sex and the presence of an AMO emerge as independent predictors of maxillary sinus pathologies; and (iv) the careful evaluation of these anatomical variations using CBCT can support multidisciplinary treatment planning in both dental and ENT practice, enhance surgical safety, and help minimize postoperative complications.

## 1. Introduction

Various anatomical variations can be observed in the nasal cavity and paranasal sinuses, including nasal septal deviation, concha bullosa, agger nasi cell, Haller cell (HC), and pneumatization of the uncinate process [1]. Some of these variations have been reported to increase susceptibility to pathology of the sinuses and nasal passages or complicate surgery in this region [2].

It has been suggested that HCs contribute to the development of maxillary sinus pathology by disrupting mucociliary clearance and causing obstructions in the osteomeatal complex [3]. First described by Albrecht von Haller, HCs are defined as ethmoidal pneumatizations of the orbital floor [4]. They are located medial to the infraorbital canal and lateral to the nasolacrimal duct [5]. This anatomical variation is often identified incidentally on cone-beam computed tomography (CBCT) scans of the paranasal sinuses and is not a pathological condition in and of itself. Large HCs have been found to cause sinusitis and headaches; however, small HCs may also contribute to these symptoms [6]. The pathophysiological significance of HCs stems from their capacity to constrict the maxillary ostium or the ethmoidal infundibulum due to their position [7]. The HC’s significance is not limited to pathological conditions. It also draws attention due to its potential to increase the risk of complications during interventional procedures, such as endoscopic sinus surgery [8].

The accessory maxillary ostium (AMO) is an anatomical variation associated with chronic sinusitis, according to several studies [9]. The AMO originates from the membranous area in the medial region of the maxilla, including the uncinate process and the inferior turbinate [10]. Although it has no physiological function when the natural maxillary sinus ostium is obstructed, the AMO has been reported in 30% of patients with chronic maxillary sinusitis and 10–20% of healthy individuals [11].

The maxillary sinus is one of the regions most frequently affected by inflammation due to its continuous exposure to irritants, and it plays a crucial role in the respiratory system [12]. The etiology of maxillary sinus pathologies is multifactorial and includes anatomical, immune, genetic, allergic, and infectious components [13]. Infections affecting these anatomical variations increase the risk of maxillary and ethmoid sinusitis. These conditions can lead to severe complications, especially when associated with maxillofacial surgery, orthognathic surgery, dental implants, maxillary sinus lift procedures, or otorhinolaryngological (ENT) interventions [14].

Conventional radiographic evaluations of the maxillary sinus only provide two-dimensional images, which are often insufficient for an accurate assessment. Therefore, CBCT is recommended for more detailed visualization due to its superior spatial resolution. CBCT enables identification of HCs, regardless of size [15]. CBCT is preferred in cases where conventional panoramic radiography is limited, especially because of its ability to clearly visualize even small HCs [16].

The prevalence of HCs varies between 10% and 45% across different populations, depending on the imaging modality and diagnostic criteria used, according to the literature [17]. Studies using CBCT have revealed these cells in many cases that were previously overlooked, strengthening the potential relationship between this anatomical variation and sinonasal diseases [18].

This study was conducted to address the gap in the literature regarding the simultaneous evaluation of variations in the maxillary sinus, such as the HC and AMO. While most existing studies examine these anatomical structures individually, this research analyzes both variations within the same patient cohort. This comprehensive approach explores their interrelationship and combined impact on maxillary sinus pathologies. Furthermore, these findings offer valuable insights into the role of anatomical variations in surgical planning and their potential to increase complication risks. In this context, the study contributes region-specific data relevant to the Turkish population and reinforces the clinical significance of a detailed preoperative radiological assessment.

The aim of this study was to evaluate the prevalence and coexistence of HCs and AMOs, as well as their relationship with maxillary ostium dimensions and sinus pathologies using CBCT, in order to provide insights relevant for preoperative assessment and risk management in dental and ENT procedures.

## 2. Materials and Methods

The study was designed as a retrospective, cross-sectional, single-center study. CBCT images obtained from patients who visited the Department of Oral, Dental, and Maxillofacial Radiology at the Zonguldak Bülent Ecevit University Faculty of Dentistry for various reasons between 2021 and 2024 were reviewed retrospectively using the digital archive. Prior to commencing the study, ethical approval was obtained from the Non-Interventional Clinical Research Ethics Committee of Zonguldak Bülent Ecevit University (date: 5 March 2025; protocol no: 2025/05-15). Informed consent was obtained from all participants before their inclusion in the study.

The study included CBCT images of patients aged 18 and older that were suitable for evaluating the maxillary sinus region, including the HC, maxillary ostium, and AMO, as well as maxillary sinus pathologies. Patients with chronic systemic diseases that could alter the sinus mucosa and images containing artifacts, cysts, tumors, or fractures were excluded. Patients with a history of maxillary sinus surgery, active infections, or malignancy were also excluded.

The power analysis for the study was performed using G*Power software (version 3.1.9.7, Kiel University, Kiel, Germany). Based on the study by Ali et al. [19] the effect size was calculated to be 0.139 for the relationship between the AMO and maxillary sinus pathologies. Using a chi-square test for goodness of fit with one degree of freedom and an alpha error probability of 0.05 (α err prob), and a power of 0.80 (1-β err prob), the required total sample size was calculated to be 407 participants (critical χ^2^ = 3.841, actual power = 0.801). To increase statistical power and ensure adequate sensitivity to detect the expected effect, this study included a total of 443 patients. CBCT images of these patients were acquired using a Veraviewepocs 3D R100 tomographic device (J. Morita Mfg. Corp., Kyoto, Japan) with the following settings: a field of view (FOV) of 8 × 10 cm, 90 kVp, 5 mA, a slice thickness of 0.5 mm, and a voxel size of 0.125 mm^3^. The CBCT images were analyzed using i-Dixel 2.0 software (J. Morita Corporation, Osaka, Japan).

The evaluation of the data was conducted once by a dentomaxillofacial radiology specialist with four years of experience. Demographic information such as patient age and sex was recorded. Images from 443 patients (886 fields, bilaterally) were evaluated.

**Figure 1:**
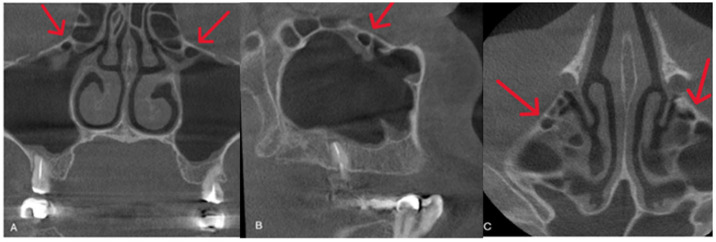
HCs are clearly identified by red arrows in each section. (**A**): coronal view; (**B**) sagittal view; (**C**) axial view.

**Figure 2:**
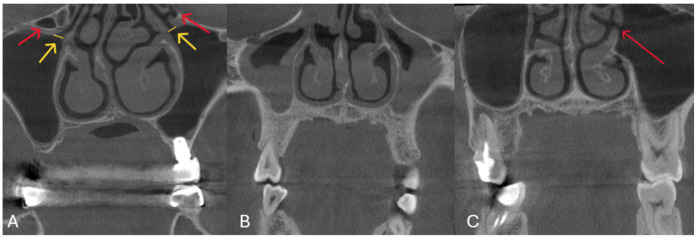
Coronal section of a CBCT image. (**A**): The maxillary ostium is indicated by yellow arrows, and HCs are indicated by red arrows and yellow lines indicate ostium width; (**B**): coronal section of a CBCT image demonstrating ostium obstruction; (**C**): coronal section of a CBCT image showing the accessory maxillary ostium indicated by a red arrow.

**Figure 3:**
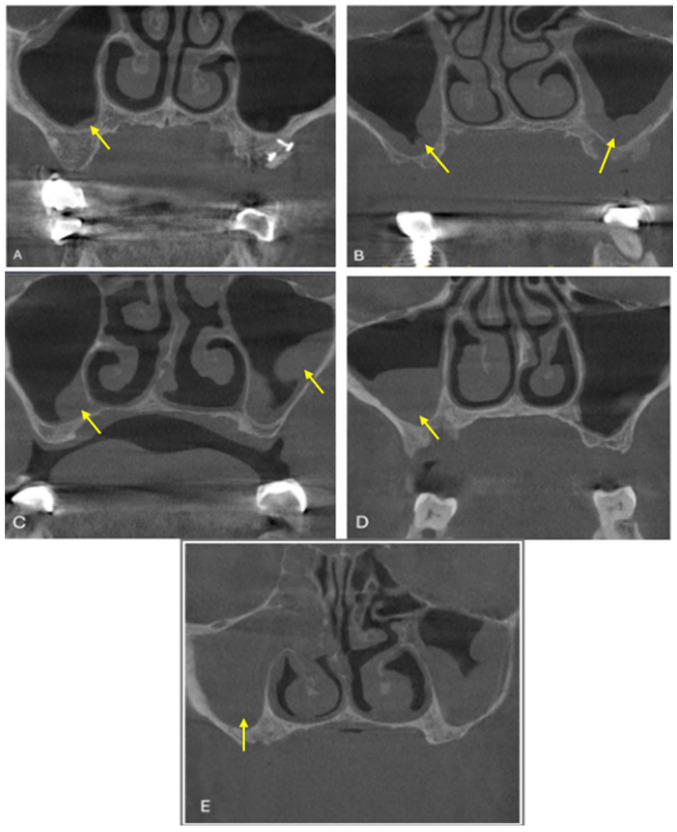
Maxillary sinus pathologies on coronal CBCT images (a yellow arrow indicates each pathology). (**A**) Localized mucosal thickening in the right maxillary sinus and a healthy left maxillary sinus; (**B**) generalized mucosal thickening in both the right and left maxillary sinuses; (**C**) polypoid mucosal thickening in the right and left maxillary sinuses; (**D**) partial opacification of the right maxillary sinus; and (**E**) total opacification of the right maxillary sinus.

**Figure 4:**
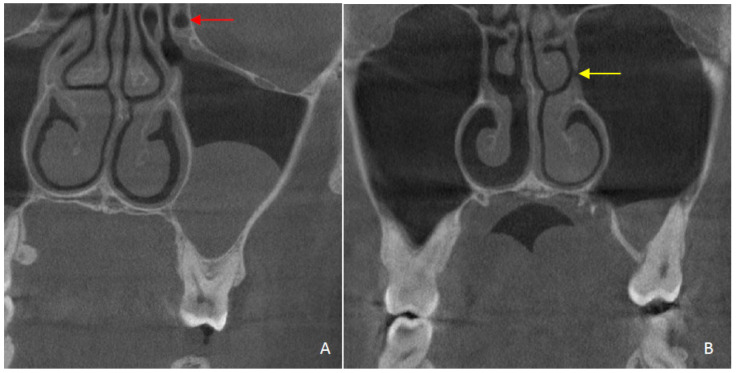
Coronal section of a CBCT scan is presented. (**A**) A Haller cell (red arrow) and a maxillary sinus pathology are observed; (**B**) the AMO (yellow arrow) and a maxillary sinus pathology are observed.

The presence and diameter of the HC were determined in the axial, coronal, and sagittal planes. Haller dimension types were classified as 0–2 mm, 2–4 mm, and greater than 4 mm. The maxillary sinus ostium, the natural drainage opening of the maxillary sinus, plays a critical role in ventilation and clearance by allowing air and mucus to pass into the nasal cavity. Ostium patency refers to the degree of openness of this passageway, which is essential for maintaining normal sinus physiology. Conversely, maxillary sinus ostium obstruction refers to the blockage of this natural opening due to causes such as inflammation, anatomical variations, nasal polyps, or septal deviation. In this study, we evaluated the maxillary sinus ostium for obstruction or narrowing and measured its width on coronal plane images. An average ostium width of 2.4 mm was determined, and values below this threshold were considered indicative of narrowing [7,9,20,21,22,23,24]. The presence of an AMO was assessed using coronal plane images. Maxillary sinus pathologies were identified and classified based on radiographic appearance as localized mucosal thickening, generalized mucosal thickening, polypoidal mucosal thickening, partial opacification, or total opacification [21]. Finally, we analyzed the relationship between the obtained data and maxillary sinus pathologies (see Figure 1, Figure 2, Figure 3 and Figure 4).

**Figure 5:**
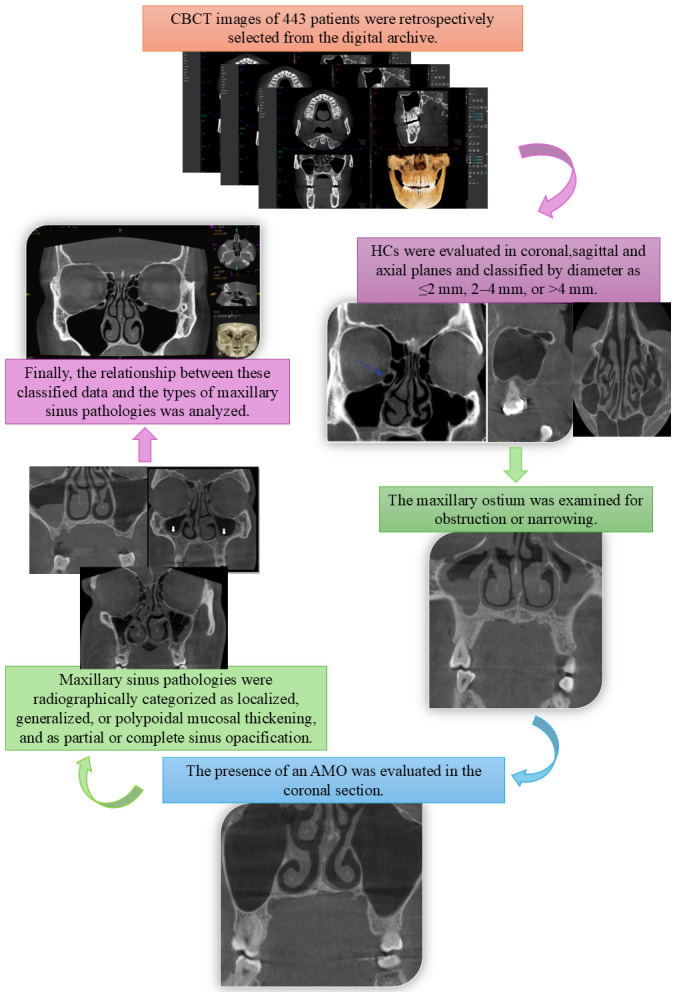
Graphical abstract illustrating the methodological process of the study.

The methodological process, research steps, and evaluation stages of our study are presented as a graphical abstract in Figure 5. This graphical abstract schematically illustrates the stages starting from the retrospective selection of CBCT images from the digital archive, the evaluation of Haller cells, the examination of the maxillary ostium for narrowing or obstruction, the identification of the presence of an accessory maxillary ostium, and the radiographic classification of maxillary sinus pathologies, as well as the analysis of the data. In this way, it aims to enable a quick and clear understanding of the research methodology and data flow.

### Statistical Analysis

Data were statistically analyzed using SPSS program (Statistical Package for the Social Sciences, version 26.0, IBM Corp., Armonk, NY, USA). The normality distribution of the data was assessed using the Kolmogorov–Smirnov test. Descriptive statistical methods, including numbers, percentages, means, standard deviations, medians, and frequencies, were used to analyze the study data. Mann–Whitney U test was used to compare continuous variables that did not follow a normal distribution. Categorical variables were compared using the Pearson Chi-square test and Fisher’s exact test. Pairwise comparisons were performed using the z-test for column proportions and the adjusted *p*-values (Bonferroni method). Intra-observer reliability was assessed by having the same examiner re-evaluate 25% of the sample two weeks after the initial assessment. For categorical variables, Cohen’s Kappa coefficient was used to evaluate agreement, while for continuous measurements, intraclass correlation coefficients (ICC) were calculated to determine consistency.

A multivariable binary logistic regression analysis was performed to evaluate independent predictors of maxillary sinus pathology. The dependent variable was the presence or absence of pathology in the right and left maxillary sinuses, while the independent variables included sex, presence of HCs (absent/present), ostium narrowing (absent/present), and the presence of an AMO (absent/present). Multicollinearity was assessed using variance inflation factor (VIF) values, which for the right maxillary sinus were 1.034 for sex, 1.118 for HCs, 1.109 for ostium narrowing, and 1.041 for AMO; for the left maxillary sinus, the corresponding VIF values were 1.012, 1.078, 1.066, and 1.014, respectively. All variables demonstrated low VIF values, indicating no evidence of multicollinearity. The results are reported as odds ratios (OR) with 95% confidence intervals (CI). The statistical significance level was set at a *p*-value of less than 0.05.

## 3. Results

The intra-observer reliability analysis demonstrated excellent agreement. For categorical variables, Cohen’s Kappa indicated a high level of consistency (κ = 0.891, *p* < 0.001). For continuous measurements, ICC values ranged from 0.887 to 0.983, confirming strong measurement reliability.

In the study sample, the prevalence of HCs was found to be 34.5% on the right side and 39.5% on the left side. The prevalence of AMO was recorded as 39.5% on the right and 34.5% on the left. Regarding the size of HCs, they were predominantly observed to measure between 2 and 4 mm on the right side, while larger cells exceeding 4 mm were more frequently identified on the left. An analysis of the distribution of maxillary sinus pathologies revealed that mucosal thickening was the most common finding on both sides. Although the mean ostium diameters were higher in females than in males bilaterally, they remained below the established reference values (Table 1).

The statistical analyses of ostium diameters according to sex, the presence of HC, AMO, and maxillary sinus pathologies are presented in Table 2. It was determined that ostium diameters were significantly smaller in the presence of HCs, ostium narrowing, and maxillary sinus pathologies (Table 2). Table 3 presents the statistical analysis of the presence of HC, ostium narrowing, AMO, maxillary sinus pathologies, HC sizes, and ostium obstruction according to sex. Maxillary sinus pathologies were more frequently observed in males on both sides. While no statistically significant difference was found between the sexes regarding the frequency of opacifications, mucosal thickenings were significantly more prevalent in males.

In cases of bilateral AMO, localized mucosal thickening occurred significantly more frequently than in cases without this anatomical variation (*p* < 0.05). Specifically, bilateral AMO was significantly associated with localized mucosal thickening, whereas no significant associations were found between AMO and other types of sinus pathology, such as generalized or polypoidal mucosal thickening, partial, or total opacification. Similarly, localized mucosal thickening was observed more frequently when both ostium were open, and partial opacification was more common in cases with ostium obstruction (Table 4).

**Table 4:** Statistical analysis results of the relationship between maxillary sinus pathologies and anatomical variables.

		Absent	Localized Mucosal Thickening	Generalized Mucosal Thickening	Polypoidal Mucosal Thickening	Partial Opacification	Total Opacification	*p* ^F^
		*n* (%)	*n* (%)	*n* (%)	*n* (%)	*n* (%)	*n* (%)	
**For Right**								
Haller cell	−	99 (66)	116 (65.2)	20 (80)	15 (50)	37 (67.3)	3 (60)	0.330
	+	52 (34)	61 (34.8)	5 (20)	15 (50)	18 (32.7)	2 (40)	
Ostium narrowing	−	97 (64.7)	115 (64.6)	15 (60)	16 (53.3)	37 (67.3)	5 (100)	0.480
	+	54 (35.3)	62 (35.4)	10 (40)	14 (46.7)	18 (32.7)	0 (0)	
Accessory maxillary ostium	−	107 (71.3) ^a^	94 (52.8) ^b^	13 (52) ^a,b^	17 (56.7) ^a,b^	32 (58.2) ^a,b^	5 (100) ^a,b^	0.005 *
	+	44 (28.7) ^a^	83 (47.2) ^b^	12 (48) ^a,b^	13 (43.3) ^a,b^	23 (41.8) ^a,b^	0 (0) ^a,b^	
Ostium obstruction	+	149 (99.3) ^a^	159 (89.3) ^b^	20 (80) ^b,c^	29 (96.7) ^a,b^	32 (58.2) ^c,d^	0 (0) ^d^	0.001 *
	−	2 (0.7) ^a^	18 (10.7) ^b^	5 (20) ^b,c^	1 (3.3) ^a,b^	23 (41.8) ^c,d^	5 (1.2) ^d^	
**For Left**								
Haller cell	−	90 (58.8)	102 (58.6)	20 (71.4)	20 (62.5)	30 (60)	6 (100)	0.336
	+	63 (41.2)	72 (41.4)	8 (28.6)	12 (37.5)	20 (40)	0 (0)	
Ostium narrowing	−	105 (68.6)	103 (59.2)	21 (75)	22 (68.8)	33 (66)	6 (100)	0.162
	+	48 (31.4)	71 (40.8)	7 (25)	10 (31.3)	17 (34)	0 (0)	
Accessory maxillary ostium	−	107 (69.9) ^a^	104 (59.8) ^a,b^	19 (67.9) ^a,b^	13 (40.6) ^b^	23 (46) ^b^	5 (83.3) ^a,b^	0.004 *
	+	46 (30.1) ^a^	70 (40.2) ^a,b^	9 (32.1) ^a,b^	19 (59.4) ^b^	27 (54) ^b^	1 (16.7) ^a,b^	
Ostium obstruction	+	151 (98.7) ^a^	165 (94.8) ^a^	18 (64.3) ^b,c^	30 (93.8) ^a,c^	27 (54) ^b^	0 (0) ^b^	0.001 *
	−	2 (1.3) ^a^	9 (5.2) ^a^	10 (35.7) ^b,c^	2 (6.3) ^a,c^	23 (46) ^b^	6 (100) ^b^	

−: indicates absent/closed; +: indicates present/open; ^F^: Fisher’s exact test; *n*: sample; %: percentage within pathologies; *p*: significance level, * *p* < 0.05; ^a,b,c,d^: There is a statistically significant difference between variables that have different top index letters in the same row.

The relationship between anatomical variations and maxillary sinus pathologies was evaluated, and the results are summarized in Table 5. Significant relationships were found between the presence of HCs and ostium narrowing, and between AMO, ostium narrowing, and maxillary sinus pathologies. The absence of HCs was associated with a significantly lower incidence of ostium narrowing. Although ostium obstruction was not observed in the absence of sinus pathology, it was occasionally present—though at a significantly lower rate—when pathology was detected. These findings suggest that sinus pathologies may contribute to ostium obstruction. Moreover, a significant association was found between the AMO and maxillary sinus pathology on both the right and left sides.

According to the results of the multivariable logistic regression analysis, sex, and the presence of an AMO were identified as independent predictors of maxillary sinus pathologies. The likelihood of both right and left maxillary sinus pathology was significantly lower in females compared to males (right OR = 0.335; left OR = 0.384; *p* < 0.001). Conversely, the presence of an AMO significantly increased the risk of pathology on both sides (right OR = 1.698, *p* = 0.018; left OR = 1.713, *p* = 0.014). Although the presence of HCs and ostium narrowing appeared to increase the risk of pathology, these associations were not statistically significant (right OR = 0.933 and 1.059; left OR = 0.852 and 1.330; *p* > 0.05). These findings suggest that, in particular, the presence of an AMO plays a clinically relevant role in determining the risk of maxillary sinus pathologies. The statistical results derived from the multivariable logistic regression analysis are presented in Table 6.

## 4. Discussion

This study used CBCT to examine the relationship between HCs, AMO, and maxillary sinus pathologies. The results were evaluated in the context of existing literature and, while corresponding with previous studies in some respects, also provide clinicians with novel insights. These results further demonstrate the value of CBCT in accurately identifying anatomical variations in the sinuses and emphasize its importance in clinical practice.

There are notable variations in the reported prevalence of HC in the literature. Valizadeh et al. [22] reported HC in 42.5% on the left side and 33.3% on the right side, while another study found a prevalence of 28%. Our findings are consistent with these values, indicating that HCs represent a clinically significant anatomical variation in the general population [5,23]. In contrast, Moshfeghi et al. [24] reported a considerably higher prevalence of 56.7%, whereas two studies focusing on the Turkish population found lower rates of 15.8% and 15.3%, respectively [13,25]. These discrepancies are thought to result from differences in population characteristics, imaging modalities, and diagnostic criteria among studies. Furthermore, in our study, no statistically significant relationship was found between the presence of HCs and maxillary sinus pathologies. Some studies have similarly reported no association [9,24], while others have suggested a positive correlation [5,7,13]. These conflicting results suggest that HCs may act as a predisposing factor but are not a direct cause of sinus pathology. In terms of size, most HCs have been reported to range between 2 and 4 mm [24], which was also the most frequent size range observed on the right side in our study. Larger cells (>4 mm) were more commonly seen on the left side and have been proposed to contribute to sinusitis by narrowing the sinus ostium and impairing mucociliary clearance [7,9,13].

Our findings indicate that the prevalence of AMO was 39.5% on the right side and 34.5% on the left. In contrast, the literature reports a wide range of prevalence rates: Yalçın et al. [13] and Özcan et al. [9] reported 27% and 24.7%, respectively, while Mahapatra et al. [23] reported 5%, and Ayyıldız and Akgunlu [26] reported 57.5%. These discrepancies are thought to result from differences in methodological approaches, demographic characteristics, and imaging techniques. Furthermore, there is no consensus regarding the etiology of AMO; Yalçın et al. [13] suggested it develops secondary to primary ostium obstruction, whereas Mahapatra et al. [23] proposed that it may enhance sinus drainage and reduce the risk of infection. The general consensus is that AMO may contribute to chronic maxillary sinusitis [9,13]. Similarly, the regression analysis in our study indicated that the presence of AMO could be a contributing factor for sinus pathology. In contrast, the study by Türker and Bulut [27] reported only a limited and side-specific relationship. Interestingly, in cases with left-sided pathology, AMO diameters were significantly larger, suggesting a potential role of unilateral mechanical factors [27].

We observed a significant reduction in ostium size in the presence of HCs. This finding suggests that HCs may exert mechanical pressure on the ostium, which could compromise sinus drainage and increase susceptibility to pathology. While some studies, such as Kamdi et al. [28], have reported a strong association between HCs, ostium narrowing, and maxillary sinus pathologies—suggesting that HCs can constrict the infundibulum and impair mucociliary clearance, thereby predisposing patients to sinus disorders—our findings did not demonstrate a significant relationship between HCs and maxillary sinus pathologies. This indicates that although HCs may influence ostium dimensions, they do not independently predict sinus disease. A previous study also reported that the coexistence of HCs and AMOs significantly increases the prevalence of maxillary and ethmoid sinusitis, affecting adjacent sinus regions [13]. Furthermore, Razavi et al. [29] reported an increased incidence of orbital floor dehiscence in patients with HCs, which may elevate the risk of surgical complications.

Original contribution of this study is its rare focus on simultaneously evaluating both HCs and AMOs. While existing literature predominantly addresses these anatomical variants in isolation, this research offers a comprehensive approach by examining their interrelationship and collective impact on maxillary sinus pathologies. Using CBCT, which has superior spatial resolution, allowed for the accurate detection and dimensional analysis of even the smallest anatomical variations. This facilitated the anticipation of potential surgical risks [3,6,7,11,13]. These anatomical structures are important not only in dental radiology but also in ENT practice.

In cases involving maxillofacial pathologies, a collaborative evaluation between ENT specialists and dental professionals is necessary. These structures should be considered not only as localized anatomical anomalies, but also as potential clinical indicators with multisystemic relevance. To give an example, HCs may contribute to clinical conditions such as chronic rhinosinusitis and headaches [30].

The findings of this study’s multivariable logistic regression analysis indicate that sex and the presence of an AMO are independent predictors of maxillary sinus pathologies. In our study, maxillary sinus mucosal thickening was found to be more prevalent in males, a finding that has also been reported in some CBCT-based studies. Although the underlying causes of this difference are not yet fully understood, the previous literature has demonstrated associations between chronic upper airway inflammation or sinus pathologies and factors such as smoking, occupational exposure to dust, gas, and fumes, as well as environmental air pollution. Therefore, the higher prevalence observed in males may be partially explained by gender-related differences in environmental or occupational exposure. In addition, dental complications resulting from poor oral hygiene are more common in men. Therefore, the greater frequency of mucosal thickening in males in our cohort likely reflects a combination of environmental exposure and physiological variability rather than a structural predisposition [31,32]. Conversely, the presence of an AMO increased the risk of pathology on both sides; this finding supports the notion that an AMO may alter sinus drainage and affect mucociliary clearance, predisposing to chronic sinusitis. Although the presence of HCs and ostium narrowing appeared to increase the risk of pathology, these effects were not statistically significant. This suggests that these anatomical variants alone are insufficient to determine the development of pathology, but may contribute to clinical risk in combination with other factors. Clinically, the identification of an AMO should be carefully considered in risk assessment and surgical planning for sinus pathologies. The findings underscore the importance of a multidisciplinary approach and highlight that considering these anatomical variants in preoperative evaluations in dental and ENT practice may help reduce the risk of complications [33,34,35].

One important limitation of this study is that potential odontogenic causes of maxillary sinus pathology—such as absence of sinus lift after dental implant placement, periapical lesions, periodontal disease, and edentulism—were not included as separate variables in the analyses. Although odontogenic factors are often associated with sinus pathology, some studies have reported contrary findings; for instance, Haylaz et al. [32] found no association with restorative procedures, oro-antral fistulas, or periodontal bone loss, Kıpçak et al. [36] reported a limited association only with periodontal bone loss, and Raitz et al. [37] found no significant correlation between periapical lesions and sinus mucosal changes. Additionally, sinus pathologies were classified only by absence/presence and type, without distinguishing underlying causes. All assessments were performed by a single radiologist, which may increase the risk of observer bias, and clinical factors such as smoking, recent dental procedures, and patient-reported symptoms were not considered. Maxillary sinus ostium narrowing was defined as widths below 2.4 mm, in accordance with the in vivo functional measurements reported by Aust and Drettner [38]. However, as ostium dimensions can vary depending on the measurement technique, imaging modality, and population characteristics [2,11,22], this threshold was used solely as a radiological reference.

Despite these limitations, the present study provides valuable insights into anatomical factors contributing to maxillary sinus pathology. HCs may exert pressure on the maxillary ostium, leading to ostial narrowing, impaired sinus drainage, and increased susceptibility to chronic sinusitis. Although the presence of an AMO does not independently predict pathology, it significantly increases the risk of both right and left maxillary sinus pathologies, highlighting its clinical relevance and contribution to disease development. The combined evaluation of HCs and AMOs emphasizes their potential role in the anatomically driven etiology of maxillary sinus disorders.

These anatomical variations are important not only in dental procedures, such as implant placement and sinus lift surgeries, but also in ENT practices, including endoscopic sinus and functional nasal surgeries, and the management of chronic rhinosinusitis. Given their proximity to vital structures, including the nasolacrimal duct, infraorbital nerve, and orbital floor, HCs and AMO may have broader systemic and neuro-ophthalmologic implications. Therefore, their identification should be incorporated into comprehensive, multidisciplinary evaluation protocols. Future studies should include detailed odontogenic assessments and emphasize multidisciplinary collaboration to better understand the interplay between anatomical variation, dental pathology, and sinonasal disease.

## 5. Conclusions

This study demonstrates that anatomical variations such as HCs and AMOs are not merely anatomical curiosities but have clinically significant implications. These structures can influence maxillary ostium patency and sinus drainage, potentially contributing to the development of chronic maxillary sinusitis and other sinus pathologies. Accurate identification of these variations using CBCT can enhance surgical planning and risk management in procedures such as implant placement, sinus lifts, and endoscopic sinus surgery. Incorporating the evaluation of HCs and AMOs into preoperative assessments through a multidisciplinary approach involving both dental and ENT specialists may reduce complication risks and optimize treatment outcomes.

## Figures and Tables

**Table 1:** Frequencies of variables and subgroups.

Variables	Right		Left	
	**Absent, *n* (%)**	**Present, *n* (%)**	**Absent, *n* (%)**	**Present, *n* (%)**
Haller cell	290 (65.5)	153 (34.5)	268 (60.5)	175 (39.5)
Ostium narrowing	285 (64.3)	158 (35.7)	290 (65.5)	153 (34.5)
Accessory maxillary ostium	268 (60.5)	175 (39.5)	271 (61.2)	172 (38.8)
Maxillary sinus pathology	151 (34.1)	292 (65.9)	153 (34.5)	290 (65.5)
	**Subgroups**	***n* (%)**	**Subgroups**	***n* (%)**
Haller dimension types	Absent	290 (65.5)	Absent	268 (60.5)
	0–2 mm	9 (2)	0–2 mm	6 (1.4)
	2–4 mm	74 (16.7)	2–4 mm	78 (17.6)
	>4 mm	70 (15.8)	>4 mm	91 (20.5)
	**Subgroups**	***n* (%)**	**Subgroups**	***n* (%)**
Maxillary sinus pathology types	Localized mucosal thickening	177 (40)	Localized mucosal thickening	174 (39.3)
	Generalized mucosal thickening	25 (5.6)	Generalized mucosal thickening	28 (6.3)
	Polypoidal mucosal thickening	30 (6.8)	Polypoidal mucosal thickening	32 (7.2)
	Partial opacification	55 (12.4)	Partial opacification	50 (11.3)
	Total opacification	5 (1.1)	Total opacification	6 (1.4)
	**Subgroups**	***n* (Mean ± SD)**	**Subgroups**	***n* (Mean ± SD)**
Ostium dimensions	Male	226 (2.05 ± 1.01)	Male	226 (2.10 ± 0.94)
	Female	217 (2.16 ± 0.84)	Female	217 (2.18 ± 0.86)
	**Open, *n* (%)**	**Closed, *n* (%)**	**Open, *n* (%)**	**Closed, *n* (%)**
Ostium obstruction	389 (87.8)	54 (12.2)	391 (88.3)	52 (11.7)

*n*: sample, %: percentage.

**Table 2:** Statistical analysis results of ostium dimensions according to sex and investigated anatomical variables.

	Variables	Subgroups	*n*	Mean ± SD (Median)	*p* ^M^
For Right					
	Sex	Male	226	2.05 ± 1.01 (2.38)	0.594
		Female	217	2.16 ± 0.84 (2.42)	
	Haller cell	Absent	290	2.16 ± 0.95 (2.49)	0.001 *
		Present	153	1.99 ± 0.86 (2.05)	
	Ostium narrowing	Absent	285	2.22 ± 1.11 (2.63)	0.001 *
		Present	158	1.89 ± 0.31 (1.94)	
	Accessory maxillary ostium	Absent	268	2.15 ± 0.88 (2.45)	0.516
		Present	175	2.03 ± 0.99 (2.32)	
	Maxillary sinus pathology	Absent	151	2.45 ± 0.5 (2.52)	0.001 *
		Present	292	1.92 ± 1.04 (2.21)	
For Left					
	Sex	Male	226	2.10 ± 0.94 (2.43)	0.649
		Female	217	2.18 ± 0.86 (2.42)	
	Haller cell	Absent	268	2.17 ± 0.97 (2.5)	0.002 *
		Present	175	2.09 ± 0.78 (2.21)	
	Ostium narrowing	Absent	290	2.25 ± 1.08 (2.65)	0.001 *
		Present	153	1.93 ± 0.27 (1.94)	
	Accessory maxillary ostium	Absent	271	2.21 ± 0.87 (2.46)	0.082
		Present	172	2.04 ± 0.94 (2.34)	
	Maxillary sinus pathology	Absent	153	2.43 ± 0.62 (2.49)	0.001 *
		Present	290	1.99 ± 0.98 (2.26)	

*n*: sample; SD: standard deviation; ^M^: Mann–Whitney U test; *p*: significance level; * *p* < 0.05.

**Table 3:** Statistical analysis results of anatomical variables and subgroups by sex.

Variables		Right			Left		
		Male (*n*)	Female (*n*)	*p* ^χ2^	Male (*n*)	Female (*n*)	*p* ^χ2^
Haller cell	Absent	151	139	0.542	144	124	0.157
	Present	75	78		82	93	
Ostium narrowing	Absent	150	135	0.361	151	139	0.542
	Present	76	82		75	78	
Accessory maxillary ostium	Absent	118 ^a^	150 ^b^	0.001 *	130	141	0.108
	Present	108 ^a^	67 ^b^		96	76	
Maxillary sinus pathology	Absent	49 ^a^	102 ^b^	0.001 *	54 ^a^	99 ^b^	0.001 *
	Present	177 ^a^	115 ^b^		172 ^a^	118 ^b^	
Haller dimension types	Absent	151	139	0.339 ^F^	114	124	0.138 ^F^
	0–2 mm	2	7		1	5	
	2–4 mm	39	35		41	37	
	>4 mm	34	36		40	51	
Maxillary sinus pathology types	Absent	49 ^a^	102 ^b^	0.001 *^,F^	54 ^a^	99 ^b^	0.001 *^,F^
	Localized mucosal thickening	103 ^a^	74 ^b^		94 ^a^	80 ^a^	
	Generalized mucosal thickening	19 ^a^	6 ^b^		22 ^a^	6 ^b^	
	Polypoidal mucosal thickening	18 ^a^	12 ^a^		22 ^a^	10 ^b^	
	Partial opacification	35 ^a^	20 ^b^		30 ^a^	20 ^a^	
	Total opacification	2 ^a^	3 ^a^		4 ^a^	2 ^a^	
Ostium obstruction	Open	192	197	0.061	194	197	0.106
	Closed	34	20		32	20	

*n*: sample; ^χ2^: Pearson chi-square test; ^F^: Fisher’s exact test; *p*: significance level, * *p* < 0.05; ^a,b^: There is a statistically significant difference between sexes that have different top index letters in the same row.

**Table 5:** Statistical analysis results of correlations between anatomical variables and sinus pathologies.

For Right		Ostium Narrowing			Accessory Maxillary Ostium			Maxillary Sinus Pathology			Ostium Obstruction		
		*n* (%)	*n* (%)	*p*	*n* (%)	*n* (%)	*p*	*n* (%)	*n* (%)	*p*	*n* (%)	*n* (%)	*p*
		−	+		−	+		−	+		+	−	
Haller cell	−	218 (76.5) ^a^	72 (45.6) ^b^	*	185 (69)	105 (60)	x	98 (64.9)	192 (65.8)	x	253 (65)	37 (68.5)	x
	+	67 (23.5) ^a^	86 (54.4) ^b^		83 (31)	70 (40)		53 (35.1)	100 (34.2)		136 (35)	17 (31.5)	
Ostium narrowing	−				175 (65.3)	110 (62.9)	x	97 (64.2)	188 (64.4)	x			
	+				93 (34.7)	65 (62.4)		54 (35.8)	104 (35.6)				
Accessory maxillary ostium	−							107 (70.9) ^a^	161 (55.1) ^b^	*	240 (61.7)	28 (51.9)	x
	+							44 (29.1) ^a^	131 (44.9) ^b^		149 (38.3)	26 (48.1)	
Maxillary sinus pathology	−										151 (38.8) ^a^	0 (0) ^b^	*
	+										238 (61.2) ^a^	54 (100) ^b^	
**For Left**		**Ostium narrowing**			**Accessory maxillary ostium**			**Maxillary sinus** **pathology**			**Ostium** **obstruction**		
		***n* (%)**	***n* (%)**	***p***	***n* (%)**	***n* (%)**	***p***	***n* (%)**	***n* (%)**	***p***	***n* (%)**	***n* (%)**	***p***
		**−**	**+**		**−**	**+**		**−**	**+**		**+**	**−**	
Haller cell	−	201 (69.3) ^a^	67 (43.8) ^b^	*	173 (63.8)	95 (55.2)	x	90 (58.8)	178 (61.4)	x	230 (58.8) ^a^	38 (73.1) ^b^	*
	+	89 (30.7) ^a^	86 (56.2) ^b^		98 (36.2)	77 (44.8)		63 (41.2)	112 (38.6)		161 (41.2) ^a^	14 (26.9) ^b^	
Ostium narrowing	−				180 (66.4)	110 (64)	x	105 (68.6)	185 (63.8)	x			
	+				91 (33.6)	62 (36)		48 (31.4)	105 (36.2)				
Accessory maxillary ostium	−							107 (69.9) ^a^	164 (56.6) ^b^	*	242 (61.9)	29 (55.8)	x
	+							46 (30.1) ^a^	126 (43.4) ^b^		149 (38.1)	23 (44.2)	
Maxillary sinus pathology	−										151 (38.6) ^a^	2 (3.8) ^b^	*
	+										240 (61.4) ^a^	50 96.2) ^b^	

−: indicates absent/closed; +: indicates present/open; *n:* sample; %: percentage within variables; *p*: significance; * *p* < 0.05; x: not significant; ^a,b^: There is a statistically significant difference between variables that have different top index letters in the same row.

**Table 6:** Results of the multivariable logistic regression analysis for right and left maxillary sinus pathologies.

Parameter	For Right			For Left		
	OR	95% CI	*p*	OR	95% CI	*p*
Sex	0.335	0.22–0.51	<0.001 *	0.384	0.25–0.57	<0.001 *
Haller cell	0.933	0.59–1.47	0.767	0.852	0.55–1.31	0.467
Ostium narrowing	1.059	0.67–1.66	0.805	1.33	0.85–2.07	0.212
Accessory maxillary ostium	1.698	1.09–2.63	0.018 *	1.713	1.11–2.63	0.014 *

OR: odd ratio (coefficient), 95% CI: 95% confidence Interval, *p*: significance; * *p* < 0.05.

## Data Availability

Most of the data generated or analyzed are included in the article. The remaining datasets used and/or analyzed during the current study are available from the corresponding author upon request.

## Abbreviations

The following abbreviations are used in this manuscript:

HC	Haller Cell
AMO	Accessory Maxillary Ostium
CBCT	Cone-Beam Computed Tomography
FOV	Field of View
ENT	Otolaryngological
